# Non-Equilibrium Polar Localization of Proteins in Bacterial Cells

**DOI:** 10.1371/journal.pone.0064075

**Published:** 2013-05-21

**Authors:** Saeed Saberi, Eldon Emberly

**Affiliations:** Physics Department, Simon Fraser University, Burnaby, British Columbia, Canada; Universitat Politecnica de Catalunya, Spain

## Abstract

Many proteins are observed to localize to the poles within bacterial cells. Some bacteria show unipolar localization, yet under different conditions bipolar patterns can emerge. One mechanism for spontaneous polar localization has been shown to involve the combination of protein aggregation and nucleoid occlusion. Whether the different observed patterns represent global energy minima for the cellular system remains to be determined. In this paper we show that for a model consisting only of protein aggregation along with an excluded volume effect due to the DNA polymer, that unipolar patterns are the global energy ground state regardless of protein concentration and DNA density. We extend the model to allow for proteins to be added to the cellular volume at a constant rate and show that bipolar (or multi-foci) patterns emerge as the result of the system being kinetically trapped in a local energy minimum. Lastly we also consider the situation of a growing cell that starts with a pre-existing aggregate at one of the poles and determine conditions under which either unipolar or bipolar patterns can exist at the point when it is ready to divide. This work sheds new interpretations on recently published experimental data and suggests experiments to test whether such a mechanism can drive patterning in bacteria.

## Introduction

Proteins in bacteria are observed to display a wide variety of localization patterns within the cell, from static polar localization to dynamic waves that propagate along the cell’s length. Proteins that interact with the cell membrane such as DivIVA in *Bacillus subtilis* have been shown to localize to regions where there are changes in curvature [1], [2] or as in the case of membrane bound receptors of the *Escherichia coli* chemotaxis network, to form periodic patterns due to a diffusion and capture mechanism[3]–[6]. In these cases, localization arises due to specific interactions of the protein with the membrane [7], [8]. Other experiments have shown that purely cytoplasmic protein can also localize within cells, usually forming foci at the poles [9]–[14]. In the bacteria *Caulobacter crescentus* the scaffolding protein PopZ localizes in a bipolar pattern yet when expressed in *E. coli* it tends to only localize to one pole [9]. Further experiments on PopZ [9], [10] as well as on misfolded protein [12] showed that patterning depends on the presence of the bacterial nucleoid and the existence of DNA free regions within the cell where the foci preferentially form; interactions with the membrane are not required. Depending on experimental conditions, the distribution of localization patterns from unipolar to multi-foci varied. What are the essential system parameters that determine the most likely pattern?

The above experiments revealed that some aggregating cytoplasmic proteins can be driven to localize due to the presence of the nucleoid. Recent computational work has shown how the types of patterns depend on the concentration of protein and the volume fraction of the bacterial chromosome [15]. In particular, the chromosome free regions at the poles were shown to arise because of the polymer nature of the DNA leading to spaces favorable for foci formation. The polymer was also shown to exert an entropic force that drives aggregates to form at the pole over other regions of the cell as those cost entropy of the polymer. Entropy has been shown to be a potential driving force for unmixing in confined systems [16], and recent experiments have begun to measure directly the physical nature of the confined chromosome [17]. Indeed, dynamic manipulation of the bacterial nucleoid can dramatically alter how proteins move and localize within a cell [18]. Due to the physical properties of the chromosome recent computational work showed how at certain concentrations of protein unipolar patterns could emerge while at higher concentrations bipolar patterning would be favored [15]. A simple dynamic piston compressing an interacting gas was used to explain the simulation results and provides a model for interpreting the observed results, in particular for the experiments on misfolded protein which provided a uniform distribution of aggregating protein as a starting point. However many systems have proteins added at a certain rate starting either with none or with some pre-existing pattern and it remains to be addressed how kinetics affects such patterning.

One particular system where kinetics likely influences patterning is when protein is added to a cell off of an inducible promoter. Examining the spatial patterns of a library of expressed GFP-tagged proteins in *E.coli* show that many display polar localization [19] with significant variability from strain to strain: from unipolar in one strain to multi-foci in another. How many of these patterns represent true localization of the endogenous protein or could they result from aggregation plus DNA occlusion mechanism described in the experimental and computational work? Recent experimental work has shown that proteins tagged with fluorescent proteins can generate spurious localization due to the generation of attractive molecular interactions between subunits [20]. Thus some of these tagged proteins likely may experience attractive interactions that arise solely because of the tagging. Because of this they could be driven to localize to the poles because of nucleoid occlusion. In addition to this, since in these systems protein is being expressed off high-copy plasmids the amount of protein is likely to vary from strain to strain as well as the rate at which it is expressed. How does the rate of addition affect patterning in addition to protein concentration and chromosomal density?

The role of kinetics in localization was studied in computational work on cluster formation on the membrane where it was shown how the rate of addition affects the spacing of clusters [4], with faster rates leading to more closely spaced clusters. They also examined how clusters form on growing cells with fixed concentrations of protein. Other work on patterning in the periplasmic space showed the importance of kinetic effects in determining the final steady-state pattern [21].

In this paper we extend prior work [15] by addressing the question of how the addition rate affects the patterning of aggregating cytoplasmic protein within bacteria. Are the observed patterns the ground state for the given conditions? Or do they represent kinetic traps? Here we first show that there is only one unique ground state pattern, the unipolar state, and that many of the observed patterns represent higher energy configurations. The system is not in equilibrium and kinetics drives the frequency with which a particular pattern is seen. We now report on the inclusion of an addition rate of protein and also consider growing cells where both the concentration of DNA and protein remain constant. This allows us to consider situations where there is a pre-existing pattern and how it changes as the cell grows and new protein is synthesized. Prior work [15] showed that protein concentration was the determining factor for unipolar versus bipolar patterning. Here we show that the tendency to form a bipolar pattern over a unipolar pattern could also be due entirely to how fast proteins are being added to the cell, suggesting another way to test this mechanism experimentally.

## Materials and Methods

The model that we simulate to study localization of aggregating particles inside a cell is based on the system published in Saberi et al [15]. Briefly, the system consists of a ball and spring polymer that represents the bacterial chromosome along with a certain number of interacting beads that represent the aggregating proteins all confined within a volume that has the shape of a cylindrical bacterial cell. The bacterial cell has a radius, *R*, with the midcell portion having a length *L* such that the total length of the bacteria is *2R+L*. The diameter of the beads making up the polymer is given by σ_c_ and we take it to have the value of twice the persistence length of double stranded DNA, 100 nm. For the protein particles, they have a diameter given by σ_p_, which we have chosen to be σ_p_ = 0.5 σ_c_ for the results in this paper and take it to represent a nucleated seed of aggregating protein. The number of beads making up the polymer is determined by the volume fraction of the chromosome, *f*_c_, which we take to be between 8–16% [22]. The number of protein beads is determined by their volume fraction, *f_\p_*, that we assume ranges from 0–2.5%. The size of the cell is given by *R* = 4 σ_c_ and unless otherwise specified, for fixed cell simulations *L* = 16 σ_c_ giving an aspect ratio of 3.

All beads within the confined volume interact via a Lennard-Jones potential with an interaction parameter and potential cut-off that depends on the types of the interacting particles given by,
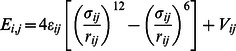



In the above, σ*_ij_* is the average diameter of the two interacting particles, *r_ij_* the distance between them, and ε*_ij_* is the interactions strength. It has three possible values: ε_cc_ for the interaction between beads on the polymer, ε_pp_ for the interaction between proteins and ε_pc_ for the interaction between polymer and protein. Both the DNA-DNA and protein-protein interactions are taken as attractive potentials with a potential cutoff equal to 2.5 σ*_ij_* and *V_ij_* = 0. The protein-polymer interaction is taken as purely repulsive and has a cutoff equal to 1.122462 σ*_ij_* and *V_ij_* = 0.25 *k_B_T*. We have the polymer-polymer interaction strength set to ε_cc_ = 0.5 *k_B_T* to favor weak condensation of the nucleoid, though the results below do not depend strongly on this. We also set the repulsive protein-polymer interaction to have ε_pc_ = 1 *k_B_T*. Beads in the polymer are also tethered with a logarithmic potential that prevents the bonds from stretching beyond a certain length. The details of the tethering potential do not alter results as a spring potential can also be used. The repulsive portion of the Lennard-Jones interaction keeps beads in the polymer from overlapping.

We simulate the model using the Metropolis algorithm. At every Monte Carlo (MC) sweep, each particle is randomly moved and its move is accepted or rejected based on the change in energy. We take the move to be uniformly distributed within a sphere of radius, *a*, that we have fixed at *a* = 0.1 σ_c_. The cell boundaries are treated as hard walls and particle moves that would cause them to leave the simulation volume are rejected. Each simulation starts with the polymer generated in a stretched linear configuration in a cell of the same length. We then gradually decrease the length of the cell, relaxing the polymer, until the cell reaches the desired start length. Then protein particles are added to the cell at a fixed rate, every certain number of MC sweeps, given by the parameter *N_add_*. Particles are added until the final desired concentration is reached.

In some of the simulations, specific initial patterns of localized proteins were required, and these were generated by adding proteins in the presence of a localizing potential. This localizing potential consists of having a force at a given position 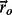 with the additional energy given by the work done by the force on each particle, 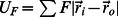. The localization potential is turned off after a certain number of MC sweeps and results collected.

We also simulate growing cells where the protein concentration is held fixed over the entire duration of the simulation. In order to do this we add to the cylindrical portion of the cell the volume occupied by a single protein every time a new protein is added to the cell. If there are *N_p_* proteins at the starting volume, *V_o_*, the amount of length that is added is given by 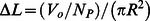. A protein is added randomly within the cell every *N_add_* MC steps, and this amount of volume is added to the cell. Since the cell grows by 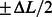, the *x* coordinates (along the length of the cell) are scaled by the fractional length change. Every time the cell is grown, the number of beads in the polymer is calculated given the polymer’s volume fraction. If this number exceeds the current number of beads in the polymer, a bead is added randomly between one of the polymers links. The existing two beads attached at that link are expanded slightly along the link direction and the new bead positioned at the midway point. The cell grows until it reaches some final aspect ratio, all the while keeping both the concentration of protein and volume fraction of polymer a constant.

## Results

### Energetics of Protein Localization

In experiments, a variety of localization patterns are observed from the bipolar patterning of PopZ protein in *C. crescentus* [9], [10], to the unipolar patterns seen for the same protein in *E. coli* [9], to a host of multiple localized foci for misfolded protein or GFP tagged protein [12], [19]. At a given concentration of aggregating protein, do these respective patterns represent the energy minima for the system?

We consider as a possible mechanism for the observed patterning the presence of the bacterial nucleoid interacting with aggregating protein (see Methods). All of the above experiments show that there is some form of attractive interaction between the proteins. The bacterial DNA acts as a region of excluded volume for the growing protein aggregate. There are no interactions with the cell membrane. (We discuss the addition of other interactions and their effect in the Discussion). In this section we examine the binding energy of the protein aggregate for different concentrations and patterns to address what is the lowest energy state of the system.

To answer the above question, we put a fixed number of aggregating protein particles into the volume to generate a fixed concentration of protein. We held the aspect ratio of the cell as well as the volume fraction of the DNA fixed for each simulation. In order to study a specific pattern of localization, we applied a weak constant localizing force at either one or several focal points within the cell volume. This force was applied for 50000 MC sweeps and then turned off. The system was then allowed to relax under zero applied force, monitoring the pattern to make sure that it stayed in the desired configuration. Simulations where the pattern dissolved were discarded. Several hundred sample configurations generated from a particular localizing force were generated and the average energy was calculated.

**Figure 1:**
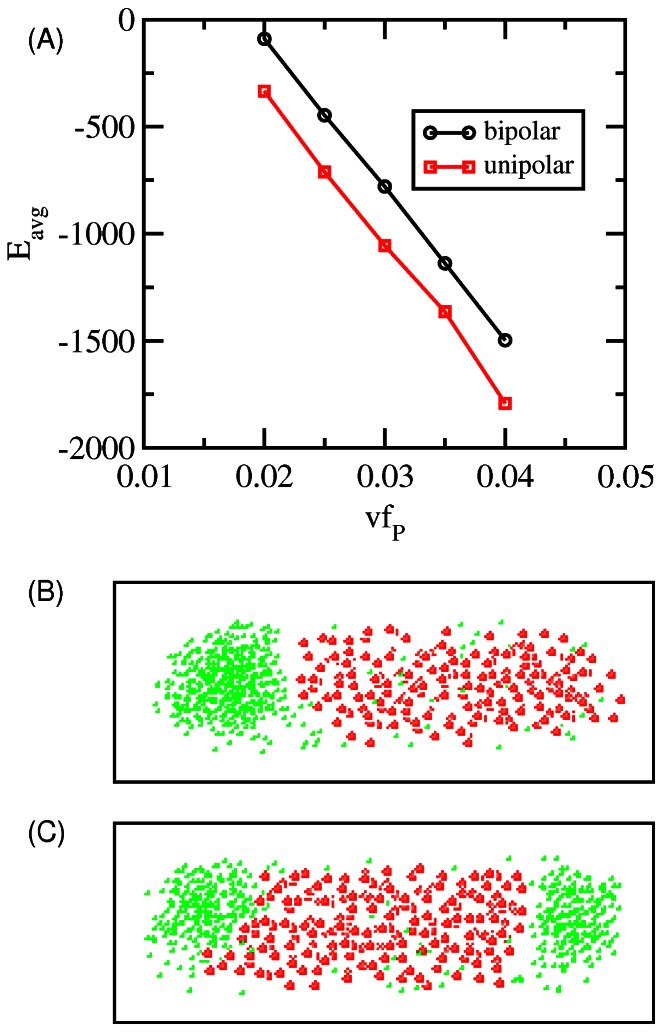
Dependence of energy on pattern. (A) Average interaction energy versus volume fraction of protein for both the unipolar and bipolar patterns. The volume fraction of chromosome was set to *f_c_* = 10% and the interaction energy *ε*_pp_ = 1.3 *k_B_T*. (B) Representative examples of unipolar and bipolar localization from simulations in (A).

The binding energy of unipolar and bipolar patterns as a function of the volume fraction of protein is shown in Fig. 1A. Below a certain protein concentration, the bipolar pattern Fig. 1C is not stable and dissolves to form a unipolar pattern, Fig. 1B, hence no data points at lower concentrations. Above this threshold concentration both patterns are stable and it is always the case that the unipolar pattern has a lower energy than the bipolar pattern at a fixed concentration. This is not surprising as having the particles distributed into two clusters leads to a greater surface energy cost than having all particles in one cluster. If we increase the interaction energy between proteins, *ε_pp_*, the same result is observed, namely that it is always energetically more favorable to have one single aggregate than two or more. Regarding how the energy varies as a function of aggregates location, we do not see much difference in the binding energy of a single aggregate whether it is at the midcell or one of the poles. As shown in our previous work [15], the drive for the cluster to form at the pole arises due to the entropic (and energetic) cost to the polymer of DNA if the cluster forms mid-cell.

Thus, based on purely energetic grounds, these results would argue that if the system could reach equilibrium, unipolar patterns should be the dominant observed pattern. Yet experimental results clearly show that for certain systems other multi-foci patterns are the most frequent. In the next two sections we show that these multi-foci patterns result because of the system being out-of-equilibrium, that either the rate at which either the concentration or cell grows can trap the system in these higher energy states.

### Protein Addition Rate Determines Localization

We now consider how the rate of adding aggregating proteins to a cell, starting with none, affects the development of localized aggregates within the cell. The type of experiments that we consider these results to be relevant to would be those in which the concentration of protein within a cell would be increasing with time, such as might be expected when expressing proteins off of an inducible promoter. We expect that if the addition rate is slow enough that the system would find its lowest energy conformation, namely a unipolar pattern. In a pure diffusion and capture mechanism, if particles are added to a cell and then diffuse, we estimate that they would get captured by a pre-existing polar aggregate if the number of time steps between additions were greater than 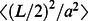 ∼10000 MC steps for the simulations below. However for faster rates, we expect that it will be possible to nucleate another seed at distances greater than the capture length of the pre-existing polar cluster. Again, the polymer will drive this newly nucleated seed to form at the other pole (or potentially between chromosomes in filamentous cells). At rates where the system would get trapped in the nearest local minima, we would expect the system to get trapped and frustrated where multiple seeds get nucleated at random locations and then grow to a size where they are no longer able to diffuse within the cell’s volume. In what follows we examine the phase space of possible patterns as a function of rate and other key parameters in the model such as protein concentration, DNA volume fraction and interaction strength.

**Figure 2:**
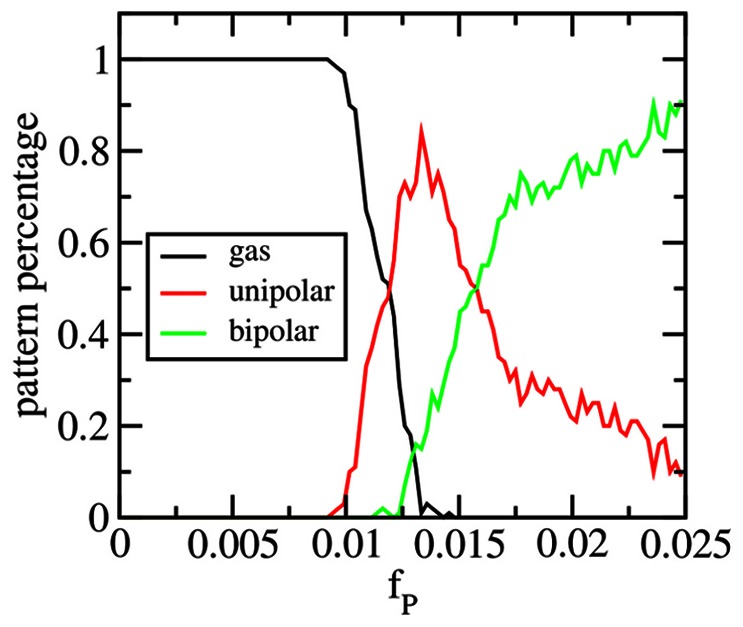
Evolution of pattern by adding particles. This shows the percentage of cells that have the given pattern (diffuse/gas = black, unipolar = red, bipolar = green) as a function of the volume fraction of protein in the cell. Proteins are added to the cell starting from zero, at a rate of *N_add_* = 1/2500 MC. All other parameters are as given in Materials and Methods and Figure 1. The averages were calculated from 50 independent simulations.

In Fig. 2 we show the percentage of cells that have a given pattern as a function of the volume fraction of protein in a cell at a given rate of protein addition. In these simulations each cell was started with no proteins and then a single protein was added at random within the volume of the cell every fixed number of MC sweeps until a final volume fraction of protein was achieved. Thus, in these simulations the protein concentration is increasing linearly in time at a rate determined by *N_add_*. There is no cell growth in these simulations, and cells have a fixed aspect ratio of 3 (we consider cell growth in the following section). In Fig. 2, proteins are added at a rate where the cell transitions from a diffuse pattern of protein to a unipolar pattern and then later at higher concentrations of protein to bipolar localization. We have defined patterns as in our previous work, based on the shape of the protein distribution as a function of cell length. A pattern is classified as diffuse, unipolar, bipolar or other, where other represents the presence of foci at locations other than that of the pole. Fig. 2 represents the expected distribution of patterns at a given rate of addition, DNA volume fraction and interaction strength. We now vary these parameters and generate phase portraits of the patterns using these calculated pattern percentages.

**Figure 3:**
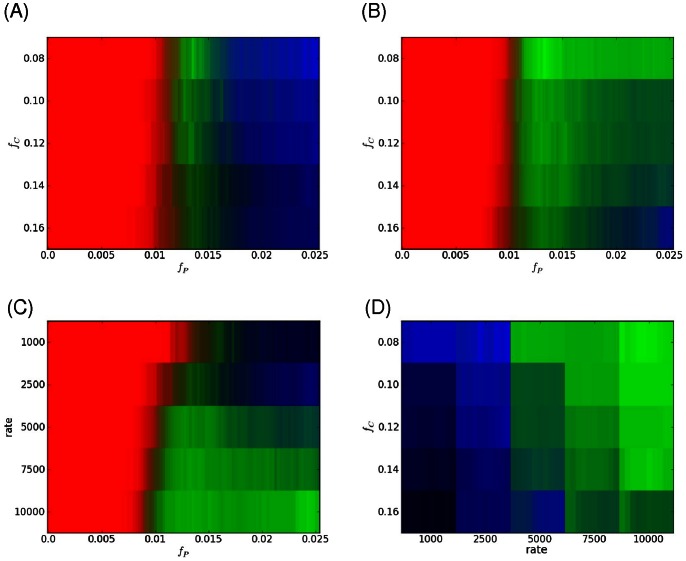
Dependence of localization on rate and volume fractions. (A, B) Evolution of the pattern of localization with increasing protein volume fraction, *f_p_*, at different chromosomal volume fractions, *f_c_*. In (A) the addition rate is *N_add_* = 1/2500 MC and (B) *N_add_* = 1/5000 MC. Here ε_pp_ = 1.2 *k_B_T*. (C) Dependence of localization pattern with increasing protein volume fraction on rate of addition for a cell with a fixed chromosomal volume fraction of *f_c_* = 14%. (D) Dependence of the final localization pattern on rate and chromosomal volume fraction (here ε_pp_ = 1.3 *k_B_T* and the final average pattern is shown for the range *f_p_* = 2.0–2.5%). We use an RGB color scheme in the figures where the patterns are colored as follows: red = gas/diffuse, green = unipolar, blue = bipolar and black = other. The color represents the fraction of cells in each pattern category (R, G, B), where the color types are as above (e.g. (1.0, 0, 0) means that 100% of cells possessed a diffuse pattern). The fractions are calculated from 50 independent cell simulations at each set of parameter values.

First we show how the resulting patterns evolve as a function of the growing protein concentration at different densities of DNA within the cell. In Fig. 3A, the pattern phase diagram is shown for a fast rate of adding proteins *N_add_* = 1/2500 MC steps. It can be seen that regardless of DNA density, at this rate of addition the pattern transitions from diffuse (red) to a short-lived unipolar situation (green), to a final bipolar pattern (blue). In Fig. 3B, a slower rate was used (*N_add_* = 1/5000 MC), and now it can be seen that at lower DNA densities, the unipolar pattern persists and continues to grow as more proteins are added to the cell. At higher DNA densities, because the polar aggregate is of smaller size its capture length does not extend the entire cell length and so at this rate it is still possible to form a bipolar pattern. So in this situation, at this given rate of adding protein, cells with lower DNA density would be unipolar while those that have a higher DNA density would be predicted to be bipolar.

Fig. 3C, D summarize the rate dependence of localization. In Fig. 3C, the phase space of localization is shown as a function of rate and protein concentration for cells possessing a fixed DNA density. At slow rates of addition (*N_add_* = 1/10000 MC steps), the unipolar pattern is seen to form at a lower protein concentration than if being added at faster rates due to the kinetics of seed formation. Once formed the unipolar pattern grows and dominates at slow rates, while at faster rates bipolar patterning is possible. The distribution of final patterns at the given final protein concentration is shown as a function of rate and DNA density in Fig. 3D. As has been noted, at the slowest rates of addition (*N_add_* = 1/10000 MC), the unipolar pattern dominates regardless of DNA density. At the fastest rate, as DNA density increases the ‘other’ pattern starts to have some significant contribution showing that the system is getting quenched into a multi-aggregate state that localizes at other locations beside the poles. In Fig. S1 we show the frequency of ‘other’ patterns as well as their average distribution, highlighting midcell formation of aggregates. At intermediate rates, depending on the DNA density, the system can be in either unipolar or bipolar patterns. These results suggest that in experiments where aggregating protein is being added to cells, multi-spot patterns are arising because the system is out of equilibrium and is getting trapped in a higher energy state than the lowest energy unipolar pattern.

**Figure 4:**
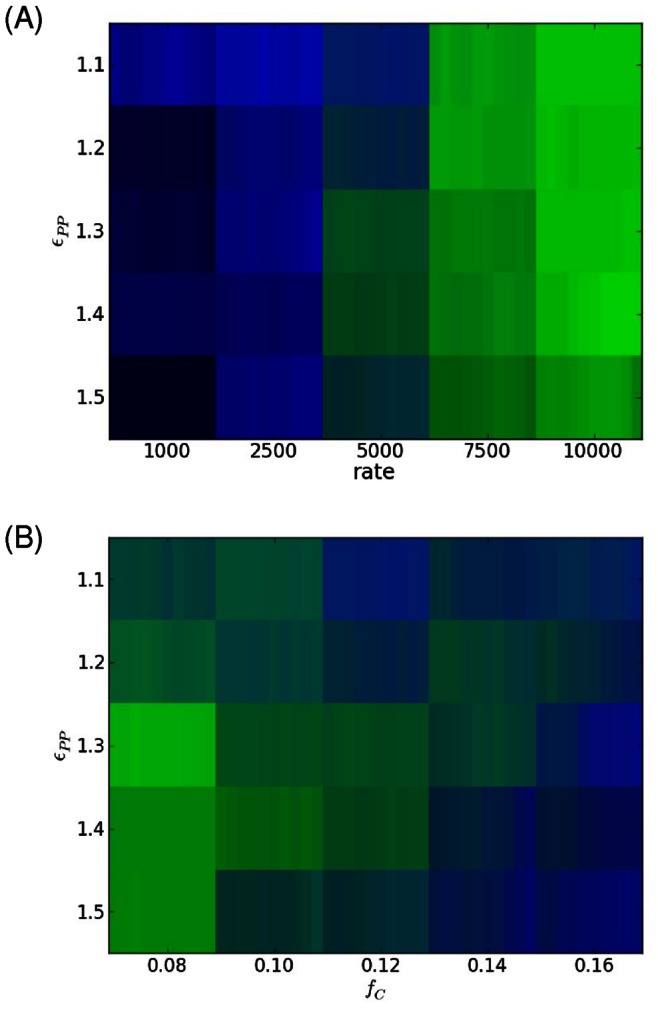
Dependence of localization on interaction strength. (A) Effect of changing rate and interaction strength on the final pattern (here *f_c_* = 12%). (B) Dependence of final pattern on interaction strength and chromosomal volume fraction (here *N_add_* = 1/5000 MC). Colors are as described in Fig. 3 and for each set of parameters the final average pattern is shown for the range *f_p_* = 2.0–2.5%.

For the last part of this section we show how the phase space of patterns changes as a function of the interaction strength between the aggregating proteins. In Fig. 4A, the final pattern distribution is shown for rate and strength of interaction at a fixed DNA density within the cell. The phase space shows the expected behavior, namely that there is a transition from bipolar (multi-foci) patterns to unipolar as the rate slows. However it shows that there are intermediate values for the interaction strength where unipolar pattern is favored at intermediate rates (*N_add_* = 1/5000 MC) whereas at lower or higher interaction strengths the bipolar pattern appears. The behavior at higher interaction strength can be understood since at a fixed rate the stronger interaction strength will favor more nucleation leading to the possibility of forming a bipolar pattern. At lower interactions strengths, it is certainly not driven by more nucleation, but rather at these intermediate rates the system has time to phase separate the aggregating proteins to the poles, allowing the bipolar pattern to eventually form. How this effect depends on DNA density is shown if Fig. 4B. As can be seen, as the DNA density increases, the values for the interaction strength where the unipolar pattern is favored shifts downwards to lower values. This can again be understood, since at higher DNA densities, the interaction energy required to nucleate a seed will be less. As has been pointed out above, at certain values for the interaction strength and rate of addition (in this case ε_pp_ = 1.3 *k_B_T*) as one goes from low to higher DNA density cell types/species, we expect to see a transition from predominantly unipolar patterns to bipolar ones. Lastly, we note that making the aggregating proteins of smaller diameter does not change the qualitative behavior described above, just the timing of aggregate formation and similarly, scaling the cell geometry also does not qualitatively change the behavior, as had been seen previously in [15].

### Protein Localization in Growing Cells

The preceding section was based on cells of a fixed size where the protein concentration increases linearly in time and the cells started with no formed aggregates of any kind. In this section we explore the consequences of a growing cell, similar to the work on cluster formation in the membrane of growing cells [4]. Here we fix the volume fraction of the DNA and protein, but grow the cell at a fixed rate. Thus the concentration of protein and DNA remain constant as the cell grows. We assume that a cell grows by adding length to its midcell cylindrical region as described in Methods. We start each simulation with an aggregate already existing at one of the poles to represent the old pole. Proteins are added until the desired concentration is reached under the action of a localizing force that creates an aggregate at one of the poles. The force is turned off and then the cells are grown as described in Methods.

**Figure 5:**
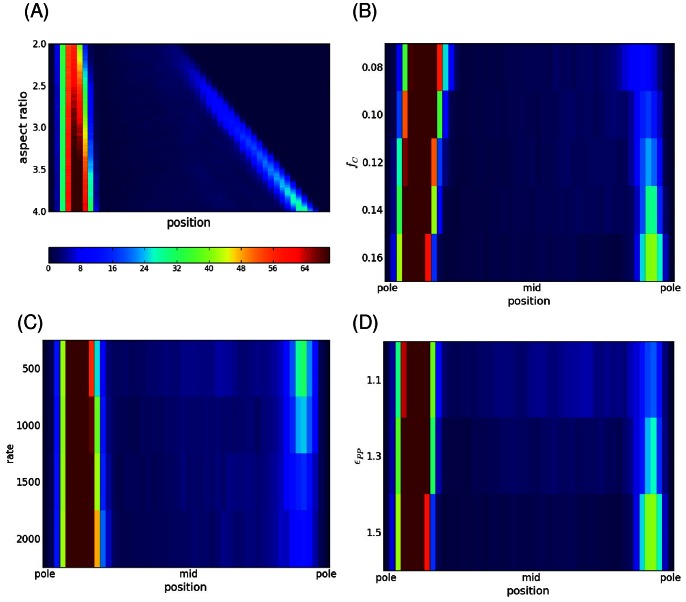
Localization patterns in growing cells. (A) Average spatial distribution of aggregating protein in growing cell – aspect ratio increases with time. The color represents the amount of protein at the given position along the cells length. Each cell starts with an aggregate at the left pole. As the cell grows an new aggregate forms at the other pole. Here the volume fraction of DNA was *f_c_* = 16%, protein amount *f_P_* = 2.25%, *N_add_* = 1/1500 MC and ε_pp_ = 1.5 *k_B_T*. (B) Dependence of spatial pattern for cells with aspect ratio = 4.0 as a function of chromosomal volume fraction (here ε_pp_ = 1.5 *k_B_T* and *N_add_* = 1/2000 MC). (C) Dependence on rate for ε_pp_ = 1.3 *k_B_T* and *f_c_* = 10%. (D) Dependence of pattern on interaction strength (here *N_add_*_ = _1/2000 and *f_c_* = 16%). In each figure the spatial distribution is an average over 25 independent simulations. The color bar represents the number of particles in that cellular cross-section.

The result for growing a cell at a fixed rate with a fixed concentration of protein and DNA is shown in Fig. 5a. What is shown is the average density of protein as a function of cell length as the cell changes its length (this is the average over 50 simulations of a growing cell for fixed parameters). Initially there is just an aggregate at one pole, the old pole. As the cell lengthens, eventually the protein is able to nucleate another seed once the new pole gets to a length beyond the capture length of the old pole, as expected for a diffusion to capture model. This new seed then captures newly added proteins within its vicinity, continuing to grow in size. Whether this seed forms at all and its final size are determined by the growth rate, protein concentration, DNA density and interaction strength. When the cell grows and new DNA is added to the cell to keep the concentration constant, we also scale all coordinates along the length so that the size of the DNA tracks with the growing cell (see Methods). If this was not done, we found that the nucleoid would not track with the cell’s length leading to a bias towards bipolar patterning since the other pole would consistently be free of the lagging DNA. This did not seem consistent with experimental observations where the nucleoid tracks with cell growth and so in what follows the results include this scaling as a part of our Monte-carlo simulations.

In the previous section it was shown that at certain rates of addition, it was possible for cells to be predominantly unipolar at lower DNA densities compared to those with higher DNA density that favor bipolar patterns. In Fig. 5B we find the same phenomenon in growing cells and the physics is essentially the same despite the simulations being quite different. The figure shows the final density of proteins as a function of position when the cell has grown to an aspect ratio of four for different DNA densities. In the previous section where cells had a fixed length, the concentration at the unoccupied pole grows as proteins are added to the cell and with higher DNA density the proteins are more concentrated there allowing seed formation to occur once the nucleation threshold is crossed. Here in these simulations the total protein concentration is a constant, but as the cell grows, the amount of protein at the unoccupied pole also grows as they move away from the capture length of the other pole. As before, with higher DNA densities, these polar proteins will be more concentrated and can form a seed at the new pole once a certain cell length is reached. These findings are consistent with what is seen experimentally. In *E. coli* (which has a DNA volume fraction ∼ 8–10%) involving misfolded protein or PopZ [9], [12], where as they grow and divide, mother cells tend to remain unipolar, producing daughter cells that possess little or no polar aggregates. Whereas bacteria such as *C. crescentus* (that have DNA volume fractions ∼ 16–18%), aggregating proteins such as PopZ would form at the new pole forming a bipolar pattern. When the system starts with a pre-existing polar cluster the existence of other patterns is strongly reduced compared to cells that start with no cluster.

In Fig. 5C, the dependence of the localization pattern when the cell is ready to divide is shown as a function of the growth rate. Because the concentration of proteins is held fixed, the rate of addition of protein is governed solely by the growth rate of the cell. For slowly growing cells, newly added protein has sufficient time to diffuse and be captured by the existing aggregate, leading to only a single aggregate in the cell. In faster growing cells, the protein does not have enough time to diffuse the length of the growing cell and under the right conditions a seed potentially can form as shown in the figure. Lastly, we consider the effect on the final pattern by increasing the interaction strength as shown in Fig. 5D. For the concentration of protein considered, at low interaction energies, there is not enough binding energy to form a stable growing seed at the other pole. Not surprisingly, as the interaction energy increases the likelihood of a seed increases and bipolar localization occurs.

## Discussion

In this paper we have examined the kinetics of nucleoid driven localization of aggregating proteins/particles within a bacterial cell. As shown in prior work, polar localization is favored due to the entropic force exerted by the chromosome on the aggregating particles. Thus seeds are favored to form and grow at the poles. In this paper, we have shown that the equilibrium pattern should be a unipolar pattern, with bipolar or multi-foci patterns being meta-stable patterns. Here we have shown that whether a bipolar pattern emerges depends strongly on the rate of addition of particles. Such patterns result due to kinetic effects, where they exist as stable kinetic traps. A diffusion and capture mechanism governs this behavior, such that if the capture length is shorter than the cell length, then a second focus is possible at the other pole. As shown above, the kinetics strongly influences whether the system will be unipolar or bipolar with the concentration of DNA and protein serving to shift the rate at which this transition occurs. Based on our findings we can interpret some of the observed experimental results on different systems. Our results where protein is added to the cell and the concentration grows in time would be akin to expressing off of plasmids. As shown the fraction of the types of patterns seen depend on rate, and experiments show that sometimes unipolar patterns are favored where as other times bipolar. One possibility for the observed unipolar pattern is that the expressed concentration is simply too low to admit the formation of a second seed. Or it could be the result of the rate at which protein is added, being slow enough so that only the unipolar pattern persists. We would predict that a test of the model in such experiments would be to vary the rate at which protein is added to the cell which could be controlled by the promoter as well as the concentration of inducer. Faster rates, generated by higher inducer concentrations should favor bipolar patterns over experiments where the protein concentration grows slower due to lower induction where unipolar patterns would prevail. Our results from the growing cell simulations was done to connect to endogenous systems such as that of PopZ localizing within the bacteria *C. crescentus*. In such a system it is reasonable to assume that the concentration of protein is constant during cell growth and the system starts with a pre-existing pattern post-division. This lead to the main difference between the fixed cell and growing cell simulations, namely that in the growing cell we start with a pre-existing aggregate at one of the poles. Thus we start with one aggregate that would have been split into a bipolar pattern in the fixed cell simulations under certain conditions. This results in having to have faster rates than in the fixed cell simulation in order to get the capture length smaller than two cell lengths so that a second seed can form at the other pole. Increasing the protein concentration only aids the formation of the seed at the other pole slightly as the initial starting aggregate is now larger giving a longer capture length. These two effects compete with each other in the formation of the new pole’s seed. Just as in the fixed cell simulation, there were rates at which the capture length was such that a second seed could form and a new foci form at the opposite pole. We also showed that for the same rate of addition/growth that depending on the chromosomal volume fraction it was possible to get either unipolar or bipolar patterns, the latter occurring at higher chromosomal fraction. This could potentially explain the difference between expressing PopZ in *E. coli* compared to *C. crescentus*, given that the latter has higher chromosomal density and hence shows bipolar patterning whereas *E. coli* was predominantly unipolar. Experiments that either expand or condense the nucleoid on cells that have defined patterns would be a possible test of such a prediction. Other recent experiments have shown that there is a spatial organization to genes within the bacterial nucleoid, leading to the suggestion that local bursting of proteins may play an important role in patterning and growth [23]–[25]. For systems expressing protein from localized positions in the chromosome, such local bursting may aid the formation of seeds to facilitate foci formation at preferred locations rather than randomly within the cell – as might occur for proteins expressed off of high copy plasmids. Adding local bursting of proteins will be considered in future work.

The qualitative dependence of the localization of aggregating protein in the presence of a bacterial polymer does not depend strongly on the specifics of the interactions, except that they be short ranged. Other work has replaced the bacterial nucleoid with a repulsive potential between the two poles [12]. The transition between unipolar and bipolar is influenced by the mobility of the nucleoid which could be included if the nucleoid is replaced by a potential. We also note that the fluctuations of the polymer are not insignificant allowing pockets where protein seeds can form. We also considered the possibility of some form of long-range repulsion between the proteins and DNA (though we expect such interactions to be screened within the cell). Simulations showed that the system rapidly separated, with proteins localizing to the poles even without any attractive interactions between them. Experiments on the localization of misfolded protein show that they remain diffuse when they are not aggregating, going against the localization that would occur if there were longer range repulsion between the DNA and protein.

The simulations described in this paper were particle based and stochastic in nature, complementary to the work on patterning in bacterial systems using reaction-diffusion approaches [26]–[28]. For a system such as the patterns formed in the MinCDE system a potential mechanism is the formation of waves due to local activation with long range repulsion between the associated factors. Similarities can be found, namely a mechanism for activation/aggregate formation with a slightly longer range repulsion generated by the presence of the DNA. Future work will consider a continuum description of the system in terms of the dynamics of the densities of protein and DNA.

An exciting experimental system that would allow for the direct testing of the mechanisms described here is the Mother Machine [29]. Recent work has detailed the measurement of the physical properties of the bacterial chromosome using a bead to push on the trapped chromosome [17], showing it to behave like a soft piston as modeled here. We envisage that such a system could be used in conjunction with aggregating particles to examine how patterns depend on chromosal density and confinement.

## Supporting Information

Figure S1 **Non-polar patterning in cells.** (A) The fraction of patterns that are ‘other’ as a function of chromosome volume fraction and protein interaction strength for a rate *N_add_* = 1/1000 MC. Shown are the frequencies of other for the final range of protein volume fractions in each square. (B) Same as in (A) except using *N_add_* = 1/2500 MC. (C) Average distribution of particles as a function of position for ‘other’ patterns at different protein concentrations. At low concentrations, the cluster tends to form midcell. At higher concentrations the pattern consists of a midcell cluster with either one or both poles also containing a cluster.
(TIFF)
