# Supplementary figures and images for: Non-Equilibrium Polar Localization of Proteins in Bacterial Cells
Source: PLoS One. 2013 May 21;8(5):e64075. doi: 10.1371/journal.pone.0064075 (PMC3660305; doi:10.1371/journal.pone.0064075)

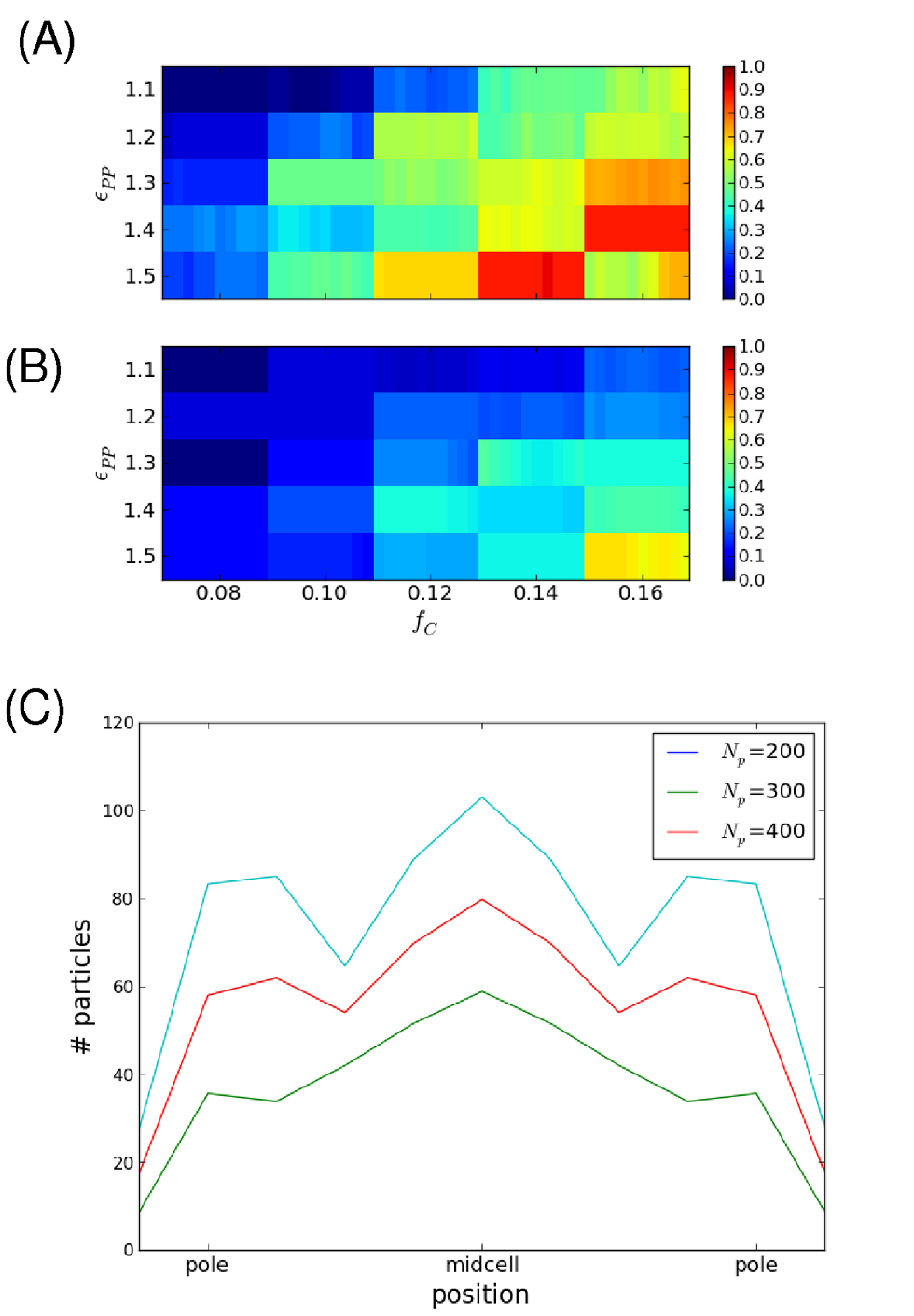

Supplement: Figure S1 — Non-polar patterning in cells. (A) The fraction of patterns that are ‘other’ as a function of chromosome volume fraction and protein interaction strength for a rate Nadd = 1/1000 MC. Shown are the frequencies of other for the final range of protein volume fractions in each square. (B) Same as in (A) except using Nadd = 1/2500 MC. (C) Average distribution of particles as a function of position for ‘other’ patterns at different protein concentrations. At low concentrations, the cluster tends to form midcell. At higher concentrations the pattern consists of a midcell cluster with either one or both poles also containing a cluster. (TIFF) [file pone.0064075.s001.tiff]
